# Cortical Brain Changes in Patients With Locked-In Syndrome Experiencing Hallucinations and Delusions

**DOI:** 10.3389/fneur.2018.00354

**Published:** 2018-05-17

**Authors:** Marco Sarà, Riccardo Cornia, Massimiliano Conson, Antonio Carolei, Simona Sacco, Francesca Pistoia

**Affiliations:** ^1^Post-Coma Intensive Rehabilitation Care Unit, San Raffaele Hospital, Cassino, Italy; ^2^Department of Biotechnological and Applied Clinical Sciences, Neurological Institute, University of L’Aquila, L’Aquila, Italy; ^3^Neuropsychology Laboratory, Department of Psychology, University of Campania Luigi Vanvitelli, Caserta, Italy

**Keywords:** locked-in syndrome, hallucinations, delusions, previsional, brain injury

## Abstract

Previous evidence suggests that hallucinations and delusions may be detected in patients with the most severe forms of motor disability including locked-in syndrome (LIS). However, such phenomena are rarely described in LIS and their presence may be underestimated as a result of the severe communication impairment experienced by the patients. In this study, we retrospectively reviewed the clinical history and the neuroimaging data of a cohort of patients with LIS in order to recognize the presence of hallucinations and delusions and to correlate it with the pontine damage and the presence of any cortical volumetric changes. Ten patients with LIS were included (5 men and 5 women, mean age 50.1 ± 14.6). According to the presence of indicators of symptoms, these patients were categorized as hallucinators (*n* = 5) or non-hallucinators (*n* = 5). MRI images of patients were analyzed using Freesurfer 6.0 software to evaluate volume differences between the two groups. Hallucinators showed a selective cortical volume loss involving the fusiform (*p* = 0.001) and the parahippocampal (*p* = 0.0008) gyrus and the orbital part of the inferior frontal gyrus (*p* = 0.001) in the right hemisphere together with the lingual (*p* = 0.01) and the fusiform gyrus (*p* = 0.01) in the left hemisphere. Moreover, a volumetric decrease of bilateral anterior portions of the precuneus was recognized in the hallucinators (right *p* = 0.01; left *p* = 0.001) as compared to non-hallucinators. We suggested that the presence of hallucinations and delusions in some LIS patients could be accounted for by the combination of a damage of the corticopontocerebellar pathways with cortical changes following the primary brainstem injury. The above areas are embedded within cortico-cortical and cortico-subcortical loops involved in self-monitoring and have been related to the presence of hallucinations in other diseases. The two main limitations of our study are the small sample of included patients and the lack of a control group of healthy individuals. Further studies would be of help to expand this field of research in order to integrate existing theories about the mechanisms underlying the generation of hallucinations and delusions in neurological patients.

## Introduction

False perceptions (hallucinations) and false beliefs (delusions) are mental phenomena representing the core of the symptomatology of schizophrenia. However, such symptoms are also frequently observed in persons with brain damage (1–3), but only rarely detected in patients with severe acquired brain injury, including those with locked-in syndrome (LIS) (4, 5). LIS is the result of a complete interruption of corticospinal, corticobulbar, and corticocerebellar pathways as a consequence of specific pontine damage usually resulting from a stenosis of the basilar artery. Patients with LIS are fully conscious but show quadriplegia, bilateral facial palsy, and anarthria. Vertical gaze and blinking are the only preserved movements and patients learn to communicate with the environment through eye-coded strategies. In the light of the above-reported damage, it might be expected that patients with LIS would have only symptoms pertaining to the motor domain. In reality, they can show additional symptoms, including emotional dysfunctions and motor imagery impairments, the pathophysiology of which is still a matter of debate (6–11). Moreover, when investigated using advanced techniques for cortical volumetric analyses, some patients show specific patterns of volumetric cortical changes beyond the initial brainstem damage (12, 13). Among non-motor symptoms, hallucinations and delusions are rarely described in LIS and their presence may be underestimated as a result of the extremely limited communication channel. In addition, patients with LIS, who are completely dependent on others for all their needs, may be reticent about sharing these experiences with the health-care professionals, even if they feel distressed about them.

In this study, we retrospectively reviewed the clinical history and the neuroimaging data of a cohort of patients with LIS in order to detect whether hallucinations and delusions were correlated to specific patterns of brain atrophy.

## Materials and Methods

### Participants

Data came from patients admitted to the Post-Coma Rehabilitative Care Unit of the San Raffaele Hospital, Cassino (Italy) in the past 10 years. Patients were only included in the study if they had a diagnosis of LIS, their clinical history had been documented in detail through medical records, and if they had been scanned using a 1.5 T MRI (Espree, Siemens AG, Erlangen) during their hospital stay. Patients with a previous history of severe neurological or psychiatric disease were excluded as well as patients treated with central nervous system active drugs. For the included patients, all clinical and neuroradiological data were retrospectively scanned. The research protocol was approved by the Internal Review Board of the University of L’Aquila (20/2017) and the study was carried out in accordance with its recommendations.

### Assessment of Clinical Data

All the clinical data of the included patients were reviewed in order to (1) identify the main biographical data and clinical characteristics of the subjects; (2) classify patients according to the Bauer classification of LIS (14); (3) categorize them into two groups based on the presence or not of hallucinations or delusions recorded in their clinical history following the brainstem damage. The Bauer classification distinguishes between a “pure” form (where the only remaining voluntary motion is vertical eye movements and blinking), an “incomplete” form (where some voluntary motor action other than eye movements is preserved) or a “total” form (complete loss of any motor output, including eye movements) (14). During the stay in the rehabilitation ward, clinical information had been collected by asking caregivers and by communicating directly with the patients using a coded communication system based on blinking and vertical eye movements. Through these movements, patients were asked to reply to closed questions (yes/no) about the presence of positive symptoms. Hallucinations were defined as the perception of visual, auditory, tactile, or olfactory stimuli in the ascertained absence of real external stimuli. Motor hallucinations were defined as an imaginary perception of a movement (for instance of a limb) in the absence of a real body movement. Delusions were defined as false beliefs based on erroneous inference about external reality that were firmly maintained by the patient despite what almost everyone else believed and despite incontestable and obvious evidence to the contrary. The presence of hallucinations was investigated through standardized screening questions in each sensory modality (15). The occurrence of delusions was mainly inferred by the reports of caregivers and further investigated through standardized screening questions (15).

In the case of positive responses to screening questions, patients were asked to better explain their experiences by using a spelling system of communication based on the selection of letters on an alphabet board through eye blinking. This allowed us to be sure about the initial detection of hallucinations and delusions and to obtain more details about individual experiences. Patients were interviewed once a week during their stay in the rehabilitation ward and considered to have had a history of hallucinations or delusions when symptoms had occurred at least once a month. The cognitive status of the patients was also documented during their stay in rehabilitation by means of the Raven’s Colored Progressive Matrices (RCPM) (16).

### Volumetric Analysis of Neuroradiological Data

As specified above, patients were only included in the study if they had undergone a 1.5 T MRI acquisition (Espree, Siemens AG, Erlangen) with a standard 8-channel birdcage head coil. Two images had been acquired for each participant and each time point. The first was a high-resolution T1-weighted image (two times), acquired using a magnetization prepared rapid acquisition gradient echo sequence (repetition time 1,590 ms; echo time 2.4 ms; flip angle 0°; matrix size 192 × 192; number of slices 160; voxel size 1 mm × 1 mm × 1 mm). The second was a T2-weighted image using a fluid attenuated inversion recovery sequence (repetition time 9,000 ms; echo time 88 ms; flip angle 0°; matrix size 384 × 512; number of slices 44).

Collected MRI images were processed and analyzed using the Freesurfer image analysis suite v 6.0 software, which is innovative software freely available online (http://surfer.nmr.mgh.harvard.edu/). Its functioning is based on an inflation algorithm, which allows to inflate the brain in order to minimize the metric distortions that can occur in structural analyses due to the natural presence of depressions and grooves in the brain. Inflation is followed by registration to a spherical atlas, parcelation of the cerebral cortex, and creation of a variety of surface based data. In the present study, for each participant, mean cortical volume values of 34 brain regions, according to Desikan parcelation (17), were calculated for both the hemispheres as the average of two values, the minimal distance from gray/white matter boundary and pial surface and *vice versa*. Each participant’s brain was morphed and registered to an average spherical surface that finely aligns sulci and gyri across them. Cortical volume values were then mapped onto this average inflated surface, thus avoiding interference of cortical folding on the visualization. Statistical maps were generated using FreeSurfer’s Query, Design, Estimate, Contrast (QDEC) interface. For each hemisphere, a general linear model of the effect of age on cortical volumes was evaluated at each vertex for male and female groups. Spatial smoothing with an isotropic kernel (FWHM = 10 mm) was applied. Data were deemed statistically significant if *p* < 0.05. False discovery rate (FDR) was corrected and tables of cluster size and location were generated.

### Additional Statistical Analysis

To confirm the results of QDEC, the mean volume of selected regions of interest was extracted for each participant and data were analyzed using the IBM SPSS Statistics 20 Software. An independent *t*-test and an analysis of covariance were used to compare the cortical volume of main areas between the two clinical groups. Correction for multiple comparisons was carried out using the Benjamin–Hochberg FDR correction in the selected areas. ANOVA was applied to evaluate the effects of the main biographical and clinical factors. For comparison between groups, the level of significance was fixed at *p* < 0.05.

## Results

Ten patients with a diagnosis of LIS were included in the study (5 men and 5 women, mean age 50.1 ± 14.6). The ventral pontine damage was a consequence of a vascular injury in nine cases and of a traumatic injury in one case. All included patients had a pure form according to the traditional classification of LIS (14). Patients did not have any previous recorded history of psychiatric symptoms and cognitive dysfunction or any additional cortical or subcortical lesions beyond the pontine damage. At the time of the interviews, all RCPM scores were within normal range after adjustment for age and education according to Italian normative studies [cutoff = 18; (16)]. A recorded history of hallucinations or delusions following the pontine damage was found for 5 of the 10 patients in the study (4 males and 1 female). Of these, a combination of visual and motor phenomena was reported in four patients, while in the fifth a history of auditory hallucinations combined with a delusional thought disorder was inferred. There were no reports of olfactory or tactile hallucinations. The median time to onset of positive symptoms was 1 month from the initial injury, with a median duration of symptoms of 4 months. As regards the content of hallucinations, most of the patients reported visual hallucinations consisting of objects or individuals, who were moving in the room wards. As regards motor hallucinations, patients were asked to describe whether they have had any unusual feelings on their own body, and most of them reported imaginary perceptions of limbs movement, which were out of their control. Finally, the delusional thought disorder, which was recognized in one patient only, was classified as a jealous delusional disorder.

Anagraphic and clinical data of the two groups are reported in Table 1: the two groups were not matched by sex (non-hallucinators = 4 females; hallucinators = 1 females), but they did not differ with respect to age (non-hallucinators: mean = 43.2, SD = 17.1; hallucinators: mean = 57, SD = 8.2; *t* = −1.626, *p* > 0.05), education (non-hallucinators: mean = 12.6, SD = 4.5; hallucinators: mean = 12.4, SD = 3.2; *t* = 0.81, *p* > 0.05), time from injury (non-hallucinators: mean = 37, SD = 8.4; hallucinators: mean = 44, SD = 1.25; *t* = −0.904, *p* > 0.05), and general cognitive functioning (non-hallucinators: mean = 24.4, SD = 1.8; hallucinators: mean = 23.8, SD = 1.9; *t* = 0.507, *p* > 0.05). Moreover, the two groups were comparable with respect to their basic MRI neuroimaging findings, as none of them showed co-existing supratentorial lesions and all had the brainstem injury, which is traditionally associated with LIS. On the contrary, the advanced volumetric analysis showed the presence of cortical differences between the two groups. The Freesurfer cluster values are shown in the Table 2. Specifically, patients experiencing hallucinations showed a larger atrophy with respect to patients not experiencing hallucinations/delusions in the fusiform gyrus (*p* = 0.001), the parahippocampal gyrus (*p* = 0.0008), and the orbital part of the inferior frontal gyrus (*p* = 0.001) in the right hemisphere together with the lingual (*p* = 0.01) and the fusiform gyrus (*p* = 0.01) in the left hemisphere. On the other hand, patients not experiencing hallucinations/delusions showed a larger atrophy in the insula and the lateral orbitofrontal cortex bilaterally (*p* ≤ 0.001 and *p* ≤ 0.0045), in the left medial orbitofrontal cortex (*p* = 0.01), in the right middle frontal and temporal regions (*p* ≤ 0.002), and in the right pars opercularis (*p* = 0.01). Moreover, the precuneus showed a volumetric decrease of bilateral anterior portions in hallucinators (right *p* = 0.01; left *p* = 0.001) and a volumetric decrease of bilateral posterior portions in non-hallucinators (right *p* = 0.02; left *p* = 0.0005).

**Table 1:** Demographic and clinical characteristics of patients.

Patients	Sex	Age at admission	Education (years)	Cause	Time from injury to admission (days)	Localization of the brainstem lesion	RCPM (raw score)	Positive symptoms
1	F	31	8	TBI	50	Pons-midbrain	25	Absent
2	F	37	17	Stroke	30	Pons	26	Absent
3	F	26	13	Stroke	40	Pons	26	Absent
4	F	56	8	Stroke	30	Pons	22	Absent
5	M	66	17	Stroke	35	Pons	23	Absent
6	M	43	17	Stroke	60	Pons-midbrain	27	Auditory hallucinations and thought delusions
7	M	62	8	Stroke	40	Pons	24	Visual/motor hallucinations
8	F	59	13	Stroke	60	Pons-medulla oblongata	22	Visual/motor hallucinations
9	M	57	13	Stroke	30	Pons	23	Visual/motor hallucinations
10	M	64	12	Stroke	30	Pons	23	Visual/motor hallucinations

*All scores were within normal range on RCPM after adjustment for age and education according to Italian normative studies [cutoff = 18; (16)]*.

*TBI: traumatic brain injury; RCPM, Raven’s colored progressive matrices*.

**Table 2:** Cluster values in FS Query, Design, Estimate, Contrast analysis, FWHM = 10, threshold of 1.31.

Cluster	Brain region	Hemisphere	Talaraich coordinates			Size	*p*-Value	
			*X*	*Y*	*Z*			
1	Insula	Left	−36.9	1.8	−5.3	864.6	0.0001	No hall < hall
2	Precuneus	Left	−6.1	−70.7	44.4	140.39	0.0005	No hall < hall
3	Paracentral	Left	−11.6	−39.3	58	102.07	0.0006	No hall < hall
4	Precentral	Left	−50.2	2	29.8	106.08	0.0009	No hall < hall
5	Precuneus	Left	−13.7	−46	49	178.86	0.0011	Hall < no hall
6	Medial orbitofrontal	Left	−6.2	20.8	20.5	151.12	0.0038	No hall < hall
7	Lateral orbitofrontal	Left	−23.5	12.5	−19.2	167.84	0.0045	No hall < hall
8	Superior parietal	Left	−15	−72.1	46.8	138.65	0.0086	No hall < hall
9	Medial orbitofrontal	Left	−7.3	41.5	−17.7	134.73	0.01	No hall < hall
10	Lateral occipital	Left	−42.6	−73.7	−9	104.94	0.0104	No hall < hall
11	Lingual	Left	−15	−66	−3.7	171.59	0.0157	Hall < no hall
12	Fusiform	Left	−32.6	−34.8	−23.1	104.11	0.017	Hall < no hall
13	Parahippocampal	Right	36.5	−38.2	−11.9	330.83	0.0008	Hall < no hall
14	Insula	Right	29.7	14.5	−12.3	103.91	0.001	No hall < hall
15	Lateral orbitofrontal	Right	18.2	13.6	−21.8	132.32	0.0011	No hall < hall
16	Pars orbitalis	Right	41.1	49.6	−7	530.71	0.0011	Hall < no hall
17	Middle-temporal	Right	54.7	−9.9	−26.3	109.93	0.0012	No hall < hall
18	Fusiform	Right	32.7	64.5	−15	653.95	0.0018	Hall < no hall
19	Caudal middle frontal	Right	38.2	7.8	44	154.3	0.0021	No hall < hall
20	Precuneus	Right	15.4	−47.2	33.3	107.71	0.0166	Hall < no hall
21	Pars opercularis	Right	34.8	15	12.7	175.54	0.0184	No hall < hall
22	Precuneus	Right	18.4	−69.9	34.9	174.11	0.0204	No hall < hall

Finally, a common feature of the recognized positive phenomena was their tendency to improve when the patients were repeatedly informed about the illusory nature of their perceptions. The symptoms spontaneously improved in all the subjects with the exception of the patient experiencing a combination of hallucinations and thought delusions. In this patient, a therapy with antipsychotic drugs was required for symptoms improvement.

## Discussion

Our findings demonstrated the presence of hallucinations/delusions in a subgroup of patients with LIS, who showed a larger atrophy in a set of brain areas as compared to LIS patients not experiencing hallucinations/delusions. Such cortical volumetric changes do not amount to a macroscopic cortical atrophy, which is generally not a feature of LIS, but refer to subtle cortical differences, which are recognizable only by means of advanced brain volumetric analysis techniques.

The strength of our study lies with the investigation of a cognitive phenomenon, which seems to be largely underestimated in patients with LIS. In our opinion, there are several reasons for the lack of consistent descriptions of positive symptoms in LIS. First, there is the traditional view that patients with an isolated pontine lesion show motor symptoms exclusively, and cannot suffer from cognitive dysfunctions unless wide additional cortical damage occurs. Second, there are the well-known difficulties experienced by caregivers and health-care professionals in establishing a communication channel with the patients. When communication is attempted, it is commonly used to investigate the most basic needs of the patients, while exploring more complex matters such as subjective mental experiences remains challenging. Finally, patients may have some reticence about sharing these symptoms as a consequence of the severe disability, which makes them completely dependent on others.

The main limitation of our study lies with the small number of patients included. As LIS is a very rare condition, 10 patients are considered a good size of sample to describe symptoms, which have not yet been systematically investigated. However, the above sample is far too small to shed lights on the existence of a causal relationship between subtle cortical changes and the presence of hallucinations/delusions in patients with LIS, thus prompting caution in drawing conclusions about the possible mechanism underlying this phenomenon.

Nevertheless, cortical changes in patients with LIS, experiencing hallucinations and delusions, involved areas, which have been previously linked to the presence of these symptoms [e.g., Ref. (1)]. Specifically, our results showed a reduced cortical volume in the fusiform and lingual regions, and in the right parahippocampal cortex, of LIS patients with positive signs. This fits with many literature findings stressing the role of both ventrolateral (fusiform) and ventromedial (lingual and parahippocampal) regions of the occipito-temporal cortex in both visual hallucinations and delusions. More in detail, the fusiform area, which is a key region of the occipito-temporal ventral visual stream, plays a pivotal role in visuoperceptual processing. Much evidence demonstrated the activation of the fusiform and lingual gyri during the recognition of objects and faces and highlighted the role of brain lesions involving this area (in particular in the right hemisphere) in neuropsychological disturbances of visual processing, such as visual agnosia and prosopagnosia [e.g., Ref. (18)]. Classical evidence on the visual processing of faces and objects has also demonstrated the involvement of the parahippocampal cortex (19, 20). Moreover, ventral visual stream areas, together with the parahippocampal region, are implicated in psychotic symptoms, both hallucinations and delusions, in patients with Alzheimer’s disease (21).

When compared with LIS non-experiencing productive phenomena, hallucinators also showed a cortical atrophy in the orbital part of the inferior frontal gyrus. Neurocognitive models of visual hallucinations have highlighted the role of a pathological interaction between defective visual brain areas and altered cortical control from prefrontal areas (22, 23). For instance, recent findings converged in demonstrating that a pattern of gray matter atrophy involving both posterior and frontal areas is implied in delusional development in patients with Alzheimer’s disease and frontotemporal dementia (24–26). On this basis, we could suggest that, in LIS patients experiencing hallucinatons/delusions, a bottom-up impairment due to alterations in posterior areas would not be effectively modulated by top-down processes in prefrontal cortex. The consequence of this would be an impaired self-monitoring, leading individuals to experience their own internal mental contents as vivid external percepts (22, 27, 28).

Patients with LIS also experienced frequent positive motor phenomena mainly represented by the perception of a limb movement in the absence of a real movement. This is consistent with literature findings showing motor awareness abnormalities as a result of various forms of brain damage (29). Here, we might speculate that the interruption of the corticocerebellar pathways, combined with cortical alterations in prefrontal cortex could have impaired the capacity to provide the system with up-to-date information about actual motor abilities (30). The co-existence of these two factors may have led to perceptions of movement where no actual movement has occurred.

Finally, alternative explanations may be proposed for the patients showing a lesion also involving the midbrain: this kind of damage has been previously reported to be associated with the development of peduncular hallucinosis and psychosis, whose pathogenesis is still debated (31, 32). All these observations suggest that the presence of hallucinations and delusions in some LIS patients can be accounted for by the combination of a damage of the corticopontocerebellar pathways with cortical changes following the primary brainstem injury. This would confirm the notion that hallucinations belong to those symptoms which arise from the pathological involvement of different structures functionally connected each other rather than being the result of the dysfunction of a single region (33).

Moreover, it is important to stress here that non-hallucinators, as compared to hallucinators, also showed signs of atrophy in a large set of anterior and posterior cortical areas. At present, we are not able to provide any tentative explanation accounting for this finding: future studies, also involving a healthy control group, are needed to precisely define the nature of cortical changes in LIS patients with and without positive symptoms.

Finally, a specific feature of the recognized hallucinations in patients with LIS is their tendency to improve when patients are repeatedly informed by health care professionals or caregivers about the illusory nature of their experiences. Compared with patients who have psychotic illnesses, patients with LIS show a greater insight about their experiences, making hallucinations and delusions more likely to diminish in response to specific management of these symptoms, as the presence of insight improves treatment compliance (34).

Further studies would be of help to expand this field of research in order to integrate existing theories about the mechanisms underlying the generation of hallucinations and delusions in neurological patients.

## Ethics Statement

The research protocol was approved by the Internal Review Board of the University of L’Aquila (number of approval: 20/2017) and the study was carried out in accordance with its recommendations.

## Author Contributions

All authors contributed to the conception of the work, to data acquisition, analysis, and interpretation and to the drafting of the paper.

## Conflict of Interest Statement

The authors declare that the research was conducted in the absence of any commercial or financial relationships that could be construed as a potential conflict of interest.
